# AI-Assisted Pharmaceutical Formulation Design: Comparative Development and Experimental Evaluation of Sustained-Release Lornoxicam Tablets

**DOI:** 10.3390/ph19071070

**Published:** 2026-07-11

**Authors:** Muthanna Abdulkarim, Waleed Bawazir, Arwa Alhaj Issa, Laian Tarboush, Amal Abbara, Gamal Mahrous, Adel Alghaith, Sally Almanasra, Khaled Suwais

**Affiliations:** 1 Department of Pharmaceutical Sciences, College of Pharmacy, Alfaisal University, P.O. Box 50927, Riyadh 11533, Saudi Arabia; arwalhajissa@gmail.com (A.A.I.); ltarboush@alfaisal.edu (L.T.); aabbara@alfaisal.edu (A.A.); 2 Department of Pharmaceutics, College of Pharmacy, King Saud University, P.O. Box 2457, Riyadh 11451, Saudi Arabia; walid.a.bawazir@gmail.com (W.B.); gmmarous@ksu.edu.sa (G.M.); afalghaith@ksu.edu.sa (A.A.); 3 College of Computer and Information Sciences, Prince Sultan University, P.O. Box 66833, Riyadh 11586, Saudi Arabia; shalmanasra@psu.edu.sa; 4 Faculty of Computer Studies, Arab Open University, P.O. Box 84901, Riyadh 11681, Saudi Arabia; khaled.suwais@arabou.edu.sa

**Keywords:** large language models, ChatGPT, DeepSeek, formulation design, extended-release matrix tablets, hydrophilic matrix systems, lornoxicam, dissolution kinetics

## Abstract

**Background/Objectives:** The integration of artificial intelligence (AI) into pharmaceutical development has the potential to accelerate early-stage formulation design. In this study, large language models (ChatGPT (GPT-4o, OpenAI) and DeepSeek (DeepSeek-R1, DeepSeek AI) were evaluated as supportive tools for the design of sustained-release lornoxicam matrix tablets. Using constrained formulation prompts and a predefined excipient space, each model generated candidate formulations intended for direct compression, with the objective of producing sustained-release systems capable of mimicking the dissolution behaviour of a commercial reference product (LOROX OD 16 mg). **Methods:** The proposed formulations were prepared experimentally and evaluated for physicochemical properties, including weight variation, hardness, friability, and drug content, as well as in vitro dissolution performance over 24 h. Dissolution profiles were compared with the reference product using similarity (f_2_) and difference (f_1_) factors, and release behaviour was further characterized using kinetic models. **Results:** All formulations demonstrated sustained-release behaviour without evidence of dose dumping. One ChatGPT-generated formulation (F3C) met the regulatory criteria for dissolution similarity to the reference product (f_1_ = 9.66, f_2_ = 71.31), while the remaining formulations showed variable release behaviour with f_2_ values ranging from 28.61 to 49.70. However, F3C exceeded the pharmacopeial friability limit marginally (1.108%), while DeepSeek formulations F5D and F6D exceeded pharmacopeial assay acceptance limits. Kinetic modelling indicated a range of transport mechanisms from anomalous diffusion to super Case II transport depending on polymer composition. **Conclusions:** Although both AI systems successfully generated experimentally viable formulations, prediction accuracy analysis showed high trend-level correlations between AI-predicted and experimental dissolution profiles. However, the magnitude of quantitative error was substantial, with RMSE values exceeding 17% and MAPE values ranging from approximately 38% to 60%. These findings indicate that the models captured general release trends but did not provide reliable quantitative dissolution predictions.

## 1. Introduction

Sustained-release oral dosage forms remain among the most widely used strategies to improve dosing convenience, maintain therapeutic exposure, and reduce peak-related adverse effects [1]. Despite decades of development experience, sustained-release formulation remains a resource-intensive activity during early stages because polymer selection, viscosity grade, and the ratio of matrix formers to fillers can strongly influence both tablet manufacturability and in vitro performance. Conventional formulation workflows therefore involve repeated screening cycles, where multiple candidate compositions are manufactured and tested to approach a target dissolution profile [2].

Artificial intelligence tools have recently attracted increasing interest as supportive technologies in pharmaceutical research [3]. In particular, large language models (LLM) can generate structured text outputs that resemble formulation hypotheses, provided that the inputs are constrained to realistic manufacturing rules and a defined excipient list [4]. Unlike classical machine learning approaches that require numeric training datasets, large language models generate recommendations through language-based reasoning informed by broad scientific corpora. This makes them potentially useful for early ideation and rapid excipient selection, but their outputs are not guaranteed to be correct and must be experimentally verified [5]. Recent reviews have highlighted the growing application of artificial intelligence technologies in solid dosage form development, including formulation optimization, process development, and prediction of pharmaceutical performance [6]. Despite the growing interest in applying LLMs to pharmaceutical development, the scientific basis for comparing different LLM systems in formulation design remains underdeveloped. It remains unclear whether different general-purpose LLMs, when provided with the same active pharmaceutical ingredient, manufacturing restrictions, and target dissolution objective, generate meaningfully different formulation strategies, and whether such differences affect product quality, dissolution similarity, and predictive accuracy.

ChatGPT and DeepSeek are selected as they are widely accessible general-purpose LLMs that are widely used for scientific reasoning. The comparison is designed as a controlled experimental assessment of whether two independent LLMs can generate scientifically plausible sustained-release lornoxicam tablet formulations when exposed to the same constrained formulation task. By experimentally preparing and evaluating all generated formulations without pre-selection or manual optimization, this study advances current knowledge by moving toward comparative experimental validation of LLM-generated pharmaceutical designs.

Lornoxicam is a non-steroidal anti-inflammatory drug belonging to the oxicam class and is used for pain and inflammatory conditions [7]. It is typically administered in immediate-release dosage forms and has a relatively short elimination half-life of approximately 3–5 h [7], which can require repeated dosing to maintain therapeutic effect. Lornoxicam is classified as a Biopharmaceutics Classification System (BCS) Class II drug, characterized by low aqueous solubility (approximately 0.15 mg/mL at pH 6.8) and high intestinal permeability [8]. Its limited solubility is an important consideration in dissolution method design and in selecting appropriate buffer conditions to ensure sink conditions during in vitro testing [8]. A sustained-release lornoxicam tablet could therefore improve adherence and provide a more convenient dosing regimen. In addition, sustained-release formulations may help reduce peak-related gastrointestinal adverse effects associated with non-steroidal anti-inflammatory drugs. However, sustained-release development for lornoxicam presents formulation challenges because hydrophilic matrix systems can exhibit complex release behaviour governed by polymer hydration, swelling, erosion, and drug diffusion [9].

The present study builds directly on our previously developed framework for AI-assisted formulation design that we validated using metformin extended-release tablets (Abdulkarim et al., manuscript submitted). Here, we extend the same concept to lornoxicam 16 mg sustained-release tablets while introducing a controlled comparison between two independent LLM systems, ChatGPT and DeepSeek. Both models are given the same prompt, excipient list, dose, manufacturing constraints, and target reference product. The objective is to determine whether different LLMs could generate experimentally viable sustained-release matrix tablet formulations and whether their outputs differed in manufacturability, dissolution similarity to the reference product, release kinetics, and quantitative prediction accuracy.

## 2. Results

### 2.1. Physical Properties and Drug Content

All AI-generated lornoxicam sustained-release formulations were successfully manufactured by direct compression and produced tablets with uniform appearance and acceptable surface characteristics. No visible defects such as capping, lamination, or excessive powder adhesion were observed during compression, indicating that the proposed excipient combinations were compatible with direct compression manufacturing [1,10].

#### 2.1.1. Weight Variation

Weight variation testing demonstrated consistent tablet mass across all formulations (Table 1). For the innovator product (LOROX OD 16 mg), twenty tablets were tested with a mean weight of 243.47 mg (range: 238.7–252.1 mg). All individual tablet weights fell within the pharmacopeial acceptance limits of ±7.5% from the mean (231.29–255.64 mg) [11,12].

ChatGPT formulations showed the following weight characteristics: F1C had a mean weight of 442.77 mg with acceptable limits of 420.63–464.90 mg (23 tablets tested); F2C had a mean of 444.23 mg with limits of 422.02–466.44 mg (23 tablets tested); and F3C had a mean of 451.90 mg with limits of 429.31–474.50 mg (21 tablets tested). All individual tablets from ChatGPT formulations met pharmacopeial weight variation requirements [11,12].

DeepSeek formulations exhibited mean weights of 402.32 mg (F4D, 24 tablets tested), 402.92 mg (F5D, 27 tablets tested), and 399.96 mg (F6D, 26 tablets tested), with corresponding acceptable ranges of 382.20–422.43 mg, 382.77–423.07 mg, and 379.96–419.96 mg, respectively. All DeepSeek formulation tablets also complied with weight variation acceptance criteria [11,12]. The observed consistency in weight indicates adequate powder flow, uniform die filling, and stable compression parameters across all AI-generated formulations [1,10].

#### 2.1.2. Hardness and Mechanical Strength

Tablet hardness measurements revealed formulation-dependent differences in mechanical strength (Table 1). The innovator product (LOROX OD 16 mg) exhibited a mean hardness of 101.1 N (range: 96.1–110.5 N). ChatGPT formulations demonstrated a wide range of hardness values. F1C showed the highest mechanical strength with individual values of 146.3, 132.2, 128.5, 86.4, and 123.5 N (mean: 123.4 N). F2C exhibited intermediate hardness with values of 104.1, 106.4, 109.1, 99.1, and 95.7 N (mean: 102.9 N). F3C showed the lowest hardness among ChatGPT formulations with values of 67.3, 66.0, 71.3, 67.9, and 71.6 N (mean: 68.8 N).

DeepSeek formulations displayed consistently higher hardness values compared to ChatGPT formulations. F4D hardness values were 136.6, 136.6, 137.9, 144.9, and 141.6 N (mean: 139.5 N). F5D showed hardness values of 143.3, 132.9, 120.8, 134.9, and 128.2 N (mean: 132.0 N). F6D exhibited values of 118.8, 99.1, 91.7, 99.1, and 106.1 N (mean: 103.0 N). All formulations achieved hardness values sufficient to withstand routine handling, packaging, and transportation. The variation in hardness across formulations likely reflects differences in polymer content, polymer type, and excipient ratios, as higher polymer concentrations and certain hydrophilic polymers are known to enhance inter-particulate bonding during compression [13].

#### 2.1.3. Friability

All AI-generated formulations, with the exception of F3C, exhibited friability values below 1%, indicating adequate mechanical resistance and compliance with pharmacopeial requirements. The reference product similarly demonstrated acceptable friability, with a value of 0.826% (Table 1).

ChatGPT formulations displayed variable friability performance. F1C exhibited a friability of 0.225% (initial weight 4.44 g, final weight 4.43 g), and F2C showed 0% friability (4.49 g unchanged). However, F3C exceeded the pharmacopeial limit with a friability of 1.108% (initial weight 4.51 g, final weight 4.46 g). DeepSeek formulations all demonstrated excellent friability results well within acceptable limits. F4D showed 0% friability (4.06 g unchanged), F5D exhibited 0.247% friability (initial 4.04 g, final 4.03 g), and F6D showed 0.249% friability (initial 4.02 g, final 4.01 g). With the exception of ChatGPT F3C, all AI-generated formulations met the pharmacopeial acceptance criterion of friability ≤ 1.0% [14], confirming adequate mechanical durability and interparticulate bonding. The elevated friability of ChatGPT F3C correlates with its lower hardness values and may reflect suboptimal binder efficiency or polymer distribution within the matrix [14,15].

#### 2.1.4. Drug Content Uniformity

Drug content analysis revealed variability in lornoxicam content among the AI-generated formulations (Table 1). Two formulations generated by DeepSeek exhibited elevated assay values exceeding the acceptable pharmacopeial limits, whereas all ChatGPT-generated formulations remained within this range.

ChatGPT formulations showed the following content results: F1C contained 15.255 mg lornoxicam (95.34% of the 16 mg label claim), F2C contained 15.32 mg (95.74%), and F3C contained 16.6 mg (103%). Both F1C and F2C fell within the pharmacopeial acceptance range of 95.0–105.0%, while F3C was within the acceptance range at 103.0% [11,12]. DeepSeek formulations exhibited greater variability in drug content. F4D contained 15.512 mg (97%), which met acceptance criteria. However, F5D and F6D showed elevated drug content values of 19.1 mg (119.38%) and 18.65 mg (116.56%), respectively, both exceeding the pharmacopeial upper limit of 105.0% [11,12].

The observed content variability, particularly in DeepSeek F5D and F6D, highlights challenges in achieving dose uniformity for low-dose formulations prepared by direct compression. These findings are consistent with reported difficulties in content uniformity when drug loading is low relative to total tablet weight, where segregation and sampling variability can significantly impact assay results [16,17]. The results underscore the necessity of experimental verification of AI-generated formulation compositions and suggest that optimization of blending procedures or incorporation of additional binders may be required for formulations showing content deviation.

### 2.2. In Vitro Dissolution Profiles

**Figure 1:**
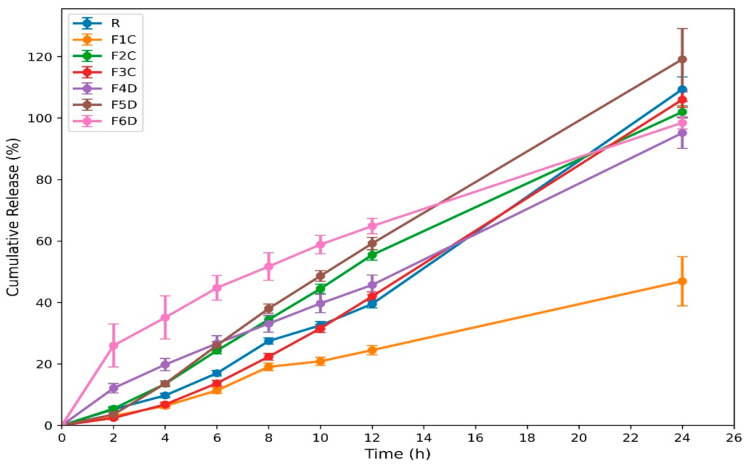
In vitro dissolution profiles of AI-generated lornoxicam extended-release matrix tablets (F1C–F6D) compared with the reference lornoxicam product in phosphate buffer (pH 6.8) using USP Apparatus II at 50 rpm and 37 ± 0.5 °C. Values represent mean percentage drug released (n = 6). Error bars indicate standard deviation.

The in vitro dissolution profiles of all AI-generated sustained-release lornoxicam tablet formulations and the innovator product (R) were evaluated over 24 h using USP Apparatus II in phosphate buffer pH 6.8 at 37 °C with 50 rpm paddle speed. Dissolution data are presented in Figure 1, and all formulations exhibited sustained-release characteristics throughout the study period, confirming that the hydrophilic polymer matrices selected by both AI models were capable of forming gel layers that controlled drug release [10,18].

The innovator product (R) demonstrated progressive drug release reaching approximately 85% at 12 h. ChatGPT formulations showed distinct release patterns: F1C exhibited the slowest release with approximately 40% drug released at 12 h, F2C displayed intermediate release behaviour, and F3C demonstrated release kinetics matching the innovator product throughout the dissolution period. Among the DeepSeek formulations, F4D and F6D exhibited sustained release profiles reaching approximately 95% and 98% cumulative release at 24 h, respectively. F5D demonstrated sustained release kinetics with a terminal cumulative release of approximately 119% at 24 h, a value attributable to its elevated measured drug content of 119.38% of label claim rather than genuine release beyond the total drug load. Minor deviations above 100% observed in other profiles are most likely attributable to normal analytical and sampling variability associated with dissolution testing.

Importantly, none of the formulations exhibited abrupt release or dose dumping at any sampling point, indicating that the AI-generated matrix designs were inherently stable and resistant to rapid structural failure under dissolution conditions. The diversity in release patterns demonstrates that both language models produced compositions spanning a rational sustained-release design space.

### 2.3. Dissolution Similarity Analysis

To further characterize dissolution behaviour relative to the innovator product, similarity factor (f_2_) and difference factor (f_1_) were calculated using the innovator as the reference (Table 2). These statistical metrics quantify the degree of similarity between each AI-generated formulation and the reference across the entire sampling period [19,20]. According to regulatory guidance, dissolution profiles are considered similar when f_1_ is less than 15 and f_2_ is between 50 and 100. Among the ChatGPT formulations, F3C demonstrated the highest similarity to the reference product (f_1_ = 9.66, f_2_ = 71.31), meeting both similarity criteria. F2C approached the similarity threshold but did not satisfy regulatory criteria (f_1_ = 25.30, f_2_ = 49.70). F1C exhibited marked deviation from the reference (f_1_ = 47.33, f_2_ = 28.61). Among the DeepSeek formulations, none met similarity criteria relative to the innovator product. F4D and F5D showed moderate deviation (f_2_ values below 50), while F6D demonstrated substantial dissimilarity (f_1_ = 67.97, f_2_ = 31.98).

These results indicate that formulations with comparable polymer architectures and excipient balances tended to produce more closely aligned release profiles relative to the innovator. Conversely, variations in polymer viscosity grade, total polymer fraction, and inclusion of additional matrix formers were associated with lower f_2_ and higher f_1_ values, reflecting measurable differences in release rate and overall profile shape. In this study, f_1_ and f_2_ values were applied to quantitatively assess the closeness of AI-generated formulations to the innovator product rather than to establish formal bioequivalence. When interpreted alongside visual inspection of dissolution curves, the similarity analysis supports the conclusion that the AI-generated formulations produced a range of sustained release behaviours requiring experimental verification [19].

### 2.4. Release Kinetics Modeling

Kinetic modeling was applied to the dissolution data to further characterize release behaviour of the AI-generated matrix systems (Table 3). Dissolution profiles were fitted to zero-order, first-order, Higuchi, and Korsmeyer–Peppas models to evaluate the extent to which different mathematical descriptions captured the observed release patterns. Zero-order fitting yielded high correlation coefficients for several formulations (R^2^ = 0.92–0.99), indicating apparent linearity of cumulative release within the studied time frame. The zero-order release constants (K_0_) ranged from 0.0333 h^−1^ for F1C to 0.0731 h^−1^ for F5D, reflecting notable differences in overall release rate among formulations. The reference product demonstrated K_0_ = 0.0700 h^−1^ with R^2^ = 0.98, comparable to F2C, F3C, and F5D, while F1C exhibited the lowest K_0_ value, consistent with its slower release profile observed in Figure 1.

First-order fitting produced more variable correlation coefficients (R^2^ = 0.75–0.99). The calculated first-order rate constants (K_1_) ranged from −0.0002 h^−1^ (F1C) to −0.0040 h^−1^ for R, F2C, F3C, and F5D. F4D and F6D showed intermediate K_1_ values of −0.0009 and −0.0012 h^−1^, respectively. The variability in first-order correlation suggests that concentration-dependent release was not uniformly dominant across all formulations. Higuchi diffusion constants (KH) ranged from 1.2610 (F1C) to 2.7868 (F5D), with correlation coefficients between R^2^ = 0.78 and 0.99. F6D demonstrated the highest correlation with the Higuchi model (R^2^ = 0.99), whereas F3C exhibited comparatively lower correlation (R^2^ = 0.78). The generally strong Higuchi fits across formulations indicate that diffusion through the hydrated matrix contributed substantially to drug release during the fitted interval [21,22].

The Korsmeyer–Peppas model showed consistently high correlation coefficients (R^2^ = 0.96–0.99) for all formulations when applied to the initial 60% of release. The calculated release exponent (n) values ranged from 0.54 to 1.53, spanning anomalous and super Case II transport regions. F4D (n = 0.81) and F6D (n = 0.54) fell within the anomalous transport range (0.45 < n < 0.89), indicating a combined influence of diffusion and polymer relaxation. In contrast, R (n = 1.17), F1C (n = 1.13), F2C (n = 1.20), F3C (n = 1.53), and F5D (n = 1.35) exhibited n values greater than 0.89, corresponding to super Case II transport behaviour within the fitted region. Overall, multiple models demonstrated acceptable fits across formulations, with differences in correlation coefficients and kinetic parameters reflecting variations in release rate and profile shape observed experimentally.

### 2.5. Statistical Analysis

Statistical analyses were conducted to further characterize relationships between dissolution similarity, kinetic parameters, and AI prediction performance. Correlation analysis, predictive accuracy metrics, and hierarchical clustering were applied to provide complementary quantitative perspectives on formulation behaviour.

#### 2.5.1. Correlation Between Dissolution Similarity and Kinetic Parameters

Pearson correlation coefficients were calculated to evaluate associations between the similarity factor (f_2_) and Korsmeyer–Peppas parameters (k_k_, n, and R^2^_kp_) (Table 4). A strong positive correlation was observed between f_2_ and the release exponent n (r ≈ 0.93), indicating that formulations exhibiting higher n values tended to demonstrate greater dissolution similarity to the reference product (Table 4). In contrast, a moderate negative correlation was observed between f_2_ and the Peppas rate constant k_k_ (r ≈ −0.59). The relationship between f_2_ and the goodness-of-fit parameter R^2^_kp_ was weak (r ≈ 0.33) (Table 4). These findings suggest that similarity in transport characteristics, reflected by the release exponent n, aligned more closely with overall dissolution profile similarity than similarity in rate constant magnitude or model fit quality. Given the limited number of formulations analyzed, correlation results were interpreted descriptively.

#### 2.5.2. AI Prediction Accuracy Assessment

Agreement between AI-predicted and experimentally measured dissolution profiles was evaluated using root mean square error (RMSE), mean absolute percentage error (MAPE), mean prediction bias (Bias), and Pearson correlation coefficients (r). The formulation-level and model-level predictive accuracy metrics are summarized in Table 5. All formulations demonstrated strong linear correlations between predicted and experimental release profiles (r = 0.95–0.99), indicating that both AI systems effectively captured the overall trend of drug release. However, quantitative deviations were evident, with RMSE values ranging from 17.9% to 25.7% and MAPE values between 38% and 60%. These findings suggest that while the AI models were capable of approximating dissolution behaviour, their predictions lacked precise quantitative accuracy. We confirm that the high Pearson correlation coefficients should not be interpreted as evidence of strong quantitative predictive performance. Correlation mainly reflects similarity in the overall increasing shape of dissolution profiles over time, where RMSE and MAPE provide a more direct indication of point-by-point prediction error. High RMSE values and large MAPE values observed across all formulations demonstrate that both LLMs had limited quantitative predictive capability. Therefore, the AI-generated dissolution profiles should be interpreted as approximate, hypothesis-generating estimates of release behavior.

ChatGPT-generated formulations exhibited slightly lower mean RMSE (≈21.6%) and MAPE (≈43%) values compared with DeepSeek-generated formulations (≈22.3% and ≈53%, respectively), while maintaining comparable Pearson correlation coefficients (≈0.98 for ChatGPT and ≈0.96 for DeepSeek). This indicates marginally better quantitative agreement for ChatGPT-generated formulations, although both systems demonstrated substantial prediction error overall. Most formulations showed a positive prediction bias, indicating systematic overestimation of cumulative drug release by the AI models, particularly at later time points. An exception was formulation F1C, which exhibited a negative bias, suggesting slight underestimation of release.

Formulation F5D demonstrated a terminal cumulative release of approximately 119% at 24 h. This value corresponds directly to its measured drug content of 119.38% of the label claim, indicating assay-driven over-recovery rather than true drug release beyond the total drug load. For formulations exceeding 100% assay, dissolution values were interpreted relative to the measured drug content to distinguish analytical variability from genuine release behaviour. Bland–Altman analysis revealed wide limits of agreement across sampling time points, indicating considerable variability between predicted and experimental dissolution values. These findings support the conclusion that AI-generated predictions should be interpreted as qualitative approximations of release behaviour rather than precise quantitative forecasts.

#### 2.5.3. Hierarchical Cluster Analysis

Hierarchical cluster analysis (HCA) was performed using standardized (z-score normalized) values of the dissolution similarity factor (f_2_) and the Korsmeyer–Peppas kinetic parameters, including the release rate constant (k_k_) and release exponent (n), to explore similarity patterns among formulations. Euclidean distance was employed as the similarity metric, and Ward’s linkage method was applied to construct the clustering hierarchy. Three distinct clusters were identified, reflecting differences in dissolution similarity and drug transport characteristics, as summarized in Table 6.

Cluster 1 comprised formulations F3C and F5D, grouped primarily on the basis of their elevated release exponent (n) values (1.53 and 1.35, respectively), consistent with relaxation-dominated or super Case II transport behaviour. It should be noted that despite their clustering together, F3C and F5D exhibited notably different f_2_ values (71.31 and 49.16, respectively), indicating that their grouping reflects mechanistic similarity rather than equivalent dissolution profile similarity to the reference product. Cluster 2 included formulations F2C and F4D, which demonstrated moderate similarity to the reference product and intermediate transport characteristics, suggesting a combination of diffusion and polymer relaxation mechanisms. Cluster 3 consisted of formulations F1C and F6D, both exhibiting lower similarity factors and comparatively lower release exponent values, indicative of reduced alignment with the reference dissolution profile. Formulations generated by both AI systems were distributed across all clusters, indicating that clustering was primarily driven by formulation composition and release behaviour rather than the origin of the AI model. Given the limited number of formulations analyzed, the clustering results were interpreted as exploratory and descriptive rather than confirmatory.

## 3. Discussion

The present study provides a comprehensive experimental evaluation of sustained release lornoxicam tablet formulations generated using large language models as supportive formulation design tools. By integrating physical characterization, dissolution testing, similarity analysis, and kinetic modelling, the work enables a multidimensional assessment of both formulation performance and the practical relevance of AI-assisted formulation ideation.

### 3.1. Manufacturability and Physical Robustness of AI-Generated Formulations

All AI-generated formulations were successfully prepared using direct compression method without observing any capping or lamination. The excipient combination suggested by both models demonstrated acceptable flow and compressibility properties. These findings confirm that the suggested matrix systems were practically suitable for standard tablet manufacturing [15].

The observed variation in hardness and friability across formulations reflects expected formulation dependent effects rather than formulation failure. Differences in polymer viscosity, polymer concentration, binders and filler type are known to affect the interparticle bonding in hydrophilic matrix systems [22,23]. Importantly, all formulations achieved sufficient mechanical strength to withstand handling and dissolution testing which confirms the feasibility of the proposed formulations.

Elevated drug content values were observed in F5D and F6D (DeepSeek formulations), exceeding acceptable limits. In contrast, all ChatGPT formulations remained within acceptable assay ranges. The content variability observed in the DeepSeek formulations may be attributed to powder segregation, or limitations associated with low dose direct compression systems [16,17]. These findings indicate that while some AI-generated formulations can achieve the basic development requirements, further formulation optimization would be necessary to achieve consistent content uniformity.

### 3.2. Interpretation of Dissolution Behaviour in Hydrophilic Matrix Systems

In this study, all formulations exhibited sustained-release behaviour without evidence of dose dumping, confirming the effective formation of hydrophilic matrix systems. Differences in release rates were consistent with established hydrophilic matrix theory. As the AI-generated formulations were based on such systems, drug release followed the fundamental sequence of events: rapid surface hydration, gel layer formation, diffusion of the dissolved drug through the swollen polymer network, and subsequent polymer relaxation and erosion. The underlying mechanism remained consistent across formulations; however, the rate of gel formation, the strength and viscosity of the hydrated barrier, and the extent of matrix porosity differed depending on composition [24]. Formulations incorporating higher proportions of high-viscosity hydrophilic polymers (F1C, F6D) exhibited slower initial release and more gradual increases in cumulative drug release over time. This behaviour is consistent with rapid polymer hydration at the tablet surface, leading to formation of dense gel barriers that restrict early drug diffusion [23,25]. As hydration progresses, the gel layer thickens and regulates drug transport through a combination of diffusion and polymer relaxation mechanisms [26].

The differences in dissolution profiles are clearly illustrated in Figure 1. Among the ChatGPT generated formulations, F1C, which contained the highest proportion of HPMC K100M (160 mg; approximately 37% of tablet weight), exhibited the most pronounced release retardation, reaching only about 47% cumulative release at 24 h. The high viscosity grade and substantial polymer fraction are expected to form a thick and cohesive gel barrier that restricts water penetration and drug diffusion, thereby accounting for the observed sustained release behaviour [19].

In contrast, F2C, which replaced the high K100M load with a combination of lower viscosity HPMC K4M and E4M CR, together with a substantial proportion of soluble Plasdone, demonstrated a markedly faster and more progressive release profile approaching complete release by 24 h. The reduced gel strength combined with increased internal porosity from soluble excipients likely facilitated more efficient diffusion at later stages [19,24]. F3C, which combined HPMC E4M with Carbopol and employed dicalcium phosphate as an insoluble filler, produced a release profile aligned with the reference product. The presence of Carbopol may have enhanced gel viscosity upon hydration, while the insoluble filler maintained matrix rigidity and limited excessive pore formation, resulting in a balanced and sustained release pattern [23,27].

Among the DeepSeek formulations, F4D and F6D were characterized by relatively high microcrystalline cellulose content and a comparatively lower total polymer fraction. Both formulations exhibited slightly faster early phase release, which may be attributed to delayed establishment of a fully cohesive gel barrier during the initial hydration stage due to reduced polymer loading. Nevertheless, both systems achieved near complete release at 24 h (approximately 95% and 98%, respectively), indicating effective matrix control. The high microcrystalline cellulose content likely contributed to structural integrity of the hydrated matrix and sustained drug release at later time points [28,29]. In contrast, F5D demonstrated sustained release kinetics overall, with a terminal cumulative release of 119% at 24 h. This value corresponds directly to its measured drug content of 119%, indicating assay driven over recovery rather than true over release [30]. The strong agreement between drug content and terminal dissolution value suggests that the apparent release above 100% is primarily a consequence of the elevated amount of drug present in the tablet rather than a failure of matrix control. Minor deviations above 100% observed in other dissolution profiles are most reasonably attributed to analytical and sampling variability associated with low-dose systems. Previous studies have reported that dissolution measurement uncertainty can exceed 6%, with sampling representing a major source of variability, while pharmacopeial guidance recognises such variation as an expected characteristic of dissolution testing [31,32].

Similarity factors f_2_ and f_1_ were used for comparative evaluation within the formulation set. These metrics were applied to assess closeness to the innovator product rather than for regulatory equivalence determination. Formulations showing higher f_2_ and lower f_1_ values shared comparable polymer composition, whereas other pairs demonstrated divergence, highlighting the sensitivity of matrix systems to polymer ratio and excipient balance (20). These findings confirm that AI-generated formulations require conventional experimental verification and optimization despite structurally reasonable compositions [33,34]. When dissolution behaviour, similarity analysis, and kinetic modelling were interpreted collectively, the results supported diffusion-controlled release with contribution from polymer relaxation processes, consistent with the expected behaviour of hydrophilic HPMC matrix systems and confirmed the scientific plausibility of the AI-generated formulation designs. It is also noteworthy that several AI-generated formulations contained disintegrants like Crosspovidone XL-10 in F1C (4.7%) and F3C (4.7%), and Croscarmellose Sodium in F2C (4.7%) and F6D (1.0%). The inclusion of disintegrants in sustained-release matrix systems is conventionally avoided, as they promote water uptake and structural disruption, which may compete with the release-retarding function of the polymer matrix. However, none of the formulations exhibited dose dumping or abrupt release, suggesting that the disintegrant concentrations selected by the AI models were insufficient to override the gel-forming capacity of the hydrophilic polymers present. In F3C specifically, the combination of HPMC E4M (23.3%), Carbopol 71G (9.3%), and a high proportion of insoluble DCP (32.6%) likely provided sufficient matrix integrity to counteract any disintegrant-driven disruption, resulting in the sustained and reference-aligned release profile observed. These findings highlight a limitation of LLM-based formulation design: the models applied disintegrants without apparent awareness of the mechanistic conflict with sustained-release matrix function, reinforcing the need for expert review of AI-generated compositions before experimental execution. This observation also highlights an important characteristic of AI-assisted formulation design. While the AI models successfully generated formulations capable of producing sustained-release behaviour, excipient selection was not always fully aligned with conventional formulation principles. Such outcomes reinforce the role of AI as a formulation ideation tool rather than a substitute for pharmaceutical expertise, with experimental evaluation remaining essential for identifying and correcting potentially inappropriate formulation choices.

One important note regarding the predictive accuracy findings, these findings should be interpreted cautiously. Although the AI-predicted dissolution profiles usually follow the same increasing trend as the experimental profiles, the absolute prediction errors were large. This indicates that the LLMs were successful in generating plausible sustained-release formulation hypotheses than in quantitatively forecasting dissolution performance. Such behaviour is expected since general-purpose LLMs do not perform mechanistic dissolution simulation and are not trained enough as formulation-specific numerical prediction models. Their outputs therefore cannot replace experimental dissolution testing, similarity analysis, or conventional formulation optimization. In the present study, the main value of the AI-assisted workflow is in the early-stage formulation ideation and structured exploration of excipient combinations.

### 3.3. Interpretation and Limitations of Kinetic Modelling

The kinetic analysis provided quantitative descriptors of release behaviour; however, mechanistic interpretation requires careful consideration of model assumptions. Although several formulations exhibited high correlation coefficients for zero-order fitting, such findings should not be interpreted as confirmation of true concentration-independent release. In hydrophilic matrix systems, apparent linearity may arise from the overlapping and gradually evolving contributions of diffusion, polymer swelling, gel layer growth, and matrix relaxation [27,35]. When these processes progress proportionally over a defined time interval, the cumulative release curve can approximate linear behaviour without strictly satisfying zero-order kinetic assumptions. Therefore, in the present study, zero-order fits were interpreted descriptively as indicators of near-linear release over the evaluated window rather than as mechanistic proof of constant-rate drug release.

The Korsmeyer–Peppas model was applied to the initial 60% of release to provide an empirical comparison of transport behaviour among formulations. The calculated release exponent (n) values spanned anomalous and super Case II transport regions. Formulations with n values in the anomalous range suggest coupled diffusion and polymer relaxation processes, whereas n values exceeding 0.89 are commonly associated with dominant polymer relaxation or matrix restructuring within the fitted interval [33,35,36,37]. Such behaviour is plausible in swellable hydrophilic matrices containing high-viscosity polymers, where significant gel formation and structural reorganization occur during hydration.

Nevertheless, the empirical nature of the Korsmeyer–Peppas equation and its sensitivity to the selected fitting range must be emphasized. Hydrophilic matrix tablets rarely conform to simplified transport assumptions throughout the entire dissolution process. Continuous structural evolution, including gel thickening, polymer disentanglement, erosion, and porosity changes, results in multi-mechanistic release behaviour. Consequently, the n values obtained in this study were interpreted comparatively rather than as definitive mechanistic assignments.

Taken together, the kinetic modelling results support a release mechanism governed by a combination of diffusion through the hydrated polymer network and polymer relaxation processes. The observation that multiple models provided acceptable fits reinforces the multi-mechanistic nature of hydrophilic matrix systems and highlights the importance of integrating kinetic modelling with dissolution profile characteristics and formulation composition when interpreting release behaviour.

### 3.4. Implications for AI-Assisted Formulation Development

From an AI perspective, the findings of this study are significant. Without access to experimental feedback or numerical training data, both ChatGPT and DeepSeek generated formulations that were manufacturable, physically robust, and capable of producing sustained-release profiles consistent with hydrophilic matrix theory. The diversity of release behaviours observed mirrors the exploratory phase of conventional formulation development.

These results support the concept that large language models can function as effective decision support tools during early formulation ideation by narrowing the experimental search space and proposing pharmaceutically reasonable starting points [38,39]. However, the observed variability in content uniformity, dissolution behaviour, and kinetic interpretation also reinforces that AI outputs cannot replace experimental optimization or pharmaceutical expertise.

The convergence of formulations from different AI models toward similar dissolution behaviour when polymer architectures aligned suggests that these models have internalized formulation principles from their training corpora. This finding has important implications for the future development of AI-assisted pharmaceutical development tools, indicating potential for knowledge extraction and application in domains with established scientific frameworks [40].

### 3.5. Statistical Perspective on Mechanistic Similarity and AI Predictive Performance

The statistical analyses provided additional quantitative insight into relationships between dissolution similarity, kinetic parameters, and AI predictive behaviour. The strong positive association observed between the similarity factor f_2_ and the Korsmeyer–Peppas release exponent n suggests that formulations exhibiting comparable transport mechanisms to the reference product also demonstrated closer overall dissolution profiles. This finding supports the interpretation that similarity in underlying release behaviour, rather than similarity in rate constant magnitude alone, is a key determinant of dissolution profile alignment in hydrophilic matrix systems.

In contrast, the weak association between f_2_ and model goodness-of-fit indicates that statistical linearity within a selected fitting range does not necessarily translate into overall profile similarity. This distinction reinforces the importance of integrating kinetic descriptors with full dissolution curve analysis rather than relying on isolated model parameters. The comparison between AI-predicted and experimental dissolution profiles further illustrates the difference between trend capture and quantitative accuracy. Although strong linear correlations were observed, substantial RMSE and MAPE values indicate that the AI systems were more effective at approximating release behaviour patterns than at predicting exact cumulative release values. This behaviour is consistent with the generative and language-based nature of large language models, which infer formulation logic rather than compute mechanistic transport equations.

Exploratory hierarchical clustering demonstrated that formulations generated by both AI systems were distributed across similarity and kinetic clusters. This observation suggests that formulation characteristics, rather than AI model identity, primarily determined release behaviour. The absence of model-specific segregation supports the view that both systems explored overlapping regions of the sustained-release design space when constrained by the same excipient framework. Given the limited number of formulations evaluated, these statistical findings should be interpreted as descriptive rather than confirmatory. Nevertheless, they provide a structured quantitative complement to the mechanistic interpretation of dissolution behaviour and offer additional perspective on the capabilities and limitations of AI-assisted formulation design.

The findings demonstrate that both ChatGPT and DeepSeek can generate formulations that are manufacturable and capable of producing sustained-release behaviour consistent with established formulation principles. The diversity of release profiles reflects the exploratory nature of early-stage formulation development. However, variability in drug content and differences in dissolution behaviour confirm that AI-generated formulations require conventional experimental validation and optimization. These results support the role of large language models as tools for early formulation ideation, while confirming that pharmaceutical expertise and process control remain essential.

Lastly, a further limitation is noted in the present study. The study did not include a random-formulation baseline. Therefore, even if only one of the six AI-generated formulations met the f_2_ similarity criterion, the data cannot determine whether the AI-assisted workflow performed better or worse than random formulation selection. Accordingly, future studies should include random, expert-designed, and statistically optimized formulation comparators to benchmark the value of LLM-assisted formulation generation extensively.

## 4. Materials and Methods

### 4.1. Materials

Lornoxicam powder was obtained from Tabuk Pharmaceuticals (Tabuk, Saudi Arabia). Dicalcium phosphate was obtained from Riedel-de Haën (Seelze, Germany). Mannitol was obtained from Qualikems Fine Chem Pvt. Ltd. (Vadodara, India). Various grades of hydroxypropyl methylcellulose (HPMC) under brand name Benecel™, including HPMC K100M DC, HPMC K4M DC, HPMC F50, HPMC E4M, HPMC A15LV, and HPMC E4M CR, were obtained as gifts from Ashland (Wilmington, DE, USA) through Brenntag Saudi Arabia Ltd., Riyadh, Saudi Arabia. Carbopol^®^ 71G NF (Lubrizol, Wickliffe, OH, USA) and colloidal silicon dioxide (Evonik, Essen, Germany) were also donated by Brenntag Saudi Arabia Ltd. Microcrystalline cellulose (Avicel PH102) and povidone K-29/32, were gifts from Sudair Pharma, Riyadh, Saudi Arabia. Croscarmellose sodium and crospovidone XL-10 were obtained from Saudi Pharmaceutical Industries (SPI), Riyadh, Saudi Arabia. Maize starch and talc were procured from Somatco, Riyadh, Saudi Arabia. Magnesium stearate (USP 43) was obtained from Anhui Sunhere Pharmaceutical Excipients Co., Ltd., Huainan, Anhui, China. Analytical grade buffer salts, including disodium hydrogen phosphate, and potassium dihydrogen phosphate, were sourced from Merck (Darmstadt, Germany). Lorox OD (16 mg sustained-release reference product) was purchased from local pharmacy, Riyadh, Saudi Arabia. Mixed cellulose ester (MCE) syringe filters (0.45 µm, 13 mm) were purchased from Merck Millipore (Burlington, MA, USA). In this study, two large language models, ChatGPT (GPT 4o, OpenAI) and DeepSeek (DeepSeek R1, DeepSeek AI), were used to generate sustained release lornoxicam tablet formulations.

### 4.2. AI-Assisted Formulation Design

To simulate early stage sustained-release formulation development, two large language models, ChatGPT and DeepSeek, were used to generate candidate sustained-release matrix tablet formulations for lornoxicam (16 mg) using a constrained prompt. The models were instructed to act as pharmaceutical formulation scientists and design sustained-release tablets by direct compression, restricted to excipients available in our laboratory, with the objective of mimicking the dissolution release profile of the reference product (LOROX OD 16 mg) under the same dissolution conditions described in Section 4.5.

To improve reproducibility of the AI-assisted workflow, the model identity, access conditions, and output-handling procedure were predefined and documented. ChatGPT-4o, OpenAI and DeepSeek-R1, DeepSeek AI were accessed in October 2025–April 2026 through official web interface. Default generation settings were used because no manual control of temperature, top-p, seed, or sampling parameters was available through the web interface.

Both models received the same prompt, the same excipient list, the same active dose of lornoxicam 16 mg, the same tablet weight restriction, the same direct-compression manufacturing constraint, and the same target objective of mimicking the dissolution behaviour of the reference product. Each model was instructed to generate exactly three sustained-release tablet formulations. The prompt was submitted once to each model, and the first complete response from each model was retained. No iterative prompt refinement, regeneration, re-ranking, or manual correction of formulation compositions was performed before experimental preparation. If a model response contained explanatory text in addition to the formulation table, only the quantitative formulation composition and the corresponding predicted dissolution values were extracted for experimental evaluation. The models were provided with the fixed active dose (lornoxicam 16 mg), a predefined list of allowable excipients, and explicit formulation constraints. The excipient list included hydrophilic polymer matrices of different viscosity grades (HPMC K100M DC, HPMC K4M DC, HPMC F50, HPMC E4M, HPMC A15LV, HPMC E4M CR, and Carbopol 71G), selected fillers and binders (microcrystalline cellulose, Plasdone K-29 32, dicalcium phosphate, and mannitol DC), and standard lubricants and glidants (Table 7). In addition, key formulation constraints such as tablet weight limits, maximum allowable lubricant percentage, and the requirement to use direct compression were specified. The AI models were instructed to propose multiple sustained-release formulations and provide brief justification for polymer selection and expected release behaviour.

**Figure 2:**
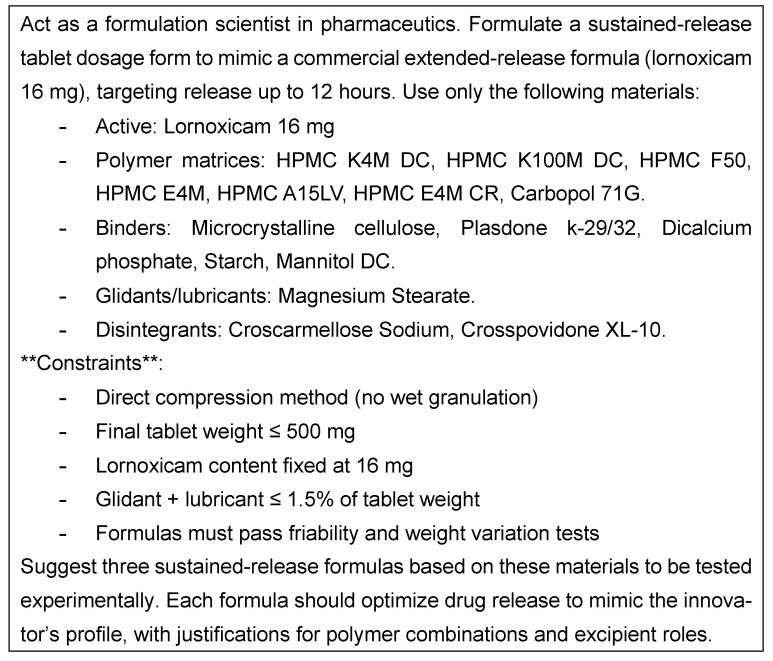
Prompt sample used to generate the three Lornoxicam formulas on both ChatGPT and DeepSeek AI models.

Three formulations were generated by ChatGPT (F1C–F3C) and three formulations were generated by DeepSeek (F4D–F6D). They were not screened, regenerated, modified, or replaced before experimental preparation. Thus, all AI-generated formulations were carried forward to laboratory manufacture and evaluation. In addition to proposing quantitative formulation compositions, each AI model was asked to predict the expected cumulative dissolution profile at each sampling time point (0.5, 1, 2, 3, 4, 6, 8, 10, 12, and 24 h), based on the provided reference product dissolution data. These AI-predicted profiles were subsequently compared with experimentally measured dissolution data to assess predictive accuracy. The qualitative and quantitative compositions of all AI-generated formulations are presented in Table 8. The prompt used for both AI models is presented in Figure 2.

### 4.3. Tablet Preparation by Direct Compression

All tablet formulations (Table 8) were produced by direct compression following standard pharmaceutical manufacturing principles [15]. Lornoxicam and excipients were accurately weighed and blended for 10 min in a Turbula^®^ mixer (Willy A. Bachofen, Switzerland) to ensure homogenous distribution of the active pharmaceutical ingredient. Magnesium stearate (0.9% *w*/*w* for ChatGPT formulations; 0.75% *w*/*w* for DeepSeek formulations) and, where applicable, colloidal silicon dioxide (0.25% *w*/*w* for DeepSeek formulations only) were added in the final step and blended for an additional three minutes to minimize the risk of over-lubrication [41].

The powder blends were compressed using a single-punch tablet press fitted with 10 mm round flat-faced punches. Each batch consisted of 200 tablets. Compression force was adjusted individually for each formulation to obtain tablets with intact surfaces, acceptable hardness (target range: 60–100 N), and minimal capping or lamination [15]. Tablets were stored in double sealed polyethylene bags containing a silica desiccant sachet at room temperature (20 to 25 °C) and protected from light prior to testing [42].

### 4.4. Physical Evaluation of Tablets

Tablets were evaluated for weight variation, diameter, hardness, friability, and drug content using pharmacopeial approaches commonly applied to compressed tablets [11,14,43]. Weight variation was assessed by weighing individual tablets and comparing each value to the batch mean. Tablet diameter was measured using a calibrated vernier caliper. Hardness was determined as tablet breaking force using a calibrated hardness tester and results were expressed in Newtons (N). Friability was assessed by subjecting tablets to rotational abrasion in a friabilator, followed by calculation of percentage weight loss. Drug content was determined by UV spectrophotometric assay of individually assayed tablets, with results expressed as percent of label claim.

#### 4.4.1. Weight Variation

Twenty tablets from each formulation were individually weighed, and the mean tablet weight was calculated. Individual tablet weights were evaluated against pharmacopeial acceptance criteria based on the average tablet weight. For tablets above 324 mg (AI-generated formulations, approximately 400 to 452 mg), not more than two tablets were permitted to deviate by more than ±5% of the mean and none by more than ±10%. For tablets with an average weight between 130 mg and 324 mg (innovator product, approximately 243 mg), the limits were ±7.5% and ±15%, respectively [11].

#### 4.4.2. Hardness

Tablet breaking force was determined for ten tablets from each formulation using a calibrated hardness tester (Erweka TBH 225 hardness tester, Langen, Germany) and expressed in Newtons (N). Mean hardness values were calculated for each formulation [44].

#### 4.4.3. Friability

Ten tablets were accurately weighed and subjected to mechanical stress in a friabilator (Erweka TA, Germany or equivalent) at 25 rpm for 100 revolutions. After testing, tablets were dedusted and reweighed. Percent weight loss was calculated according to the equation: Friability (%) = [(W_1_ − W_2_)/W_1_] × 100, where W_1_ is the initial weight and W_2_ is the final weight. A maximum weight loss of 1.0% was considered the pharmacopeial acceptance limit [14].

#### 4.4.4. Drug Content Uniformity

Ten tablets were individually assayed for lornoxicam content. Each tablet was finely powdered and quantitatively transferred to a volumetric flask. An accurately weighed quantity equivalent to the nominal dose was extracted using methanol: phosphate buffer pH 7.4 (50:50 v/v), sonicated for 15 min, filtered through a 0.45 μm membrane filter, and appropriately diluted. Lornoxicam content was determined by UV spectrophotometry at λmax = 378 nm using a validated calibration curve (linearity range: 2–20 μg/mL, R^2^ > 0.999) according to USP <905>. Drug content was expressed as percent of label claim, with acceptance limits of 95.0–105.0% [11]. All measurements were performed in triplicate unless otherwise specified, and results were expressed as mean ± standard deviation.

### 4.5. In Vitro Dissolution Study

In vitro dissolution testing was performed using USP Apparatus II (paddle method) to evaluate the release behaviour of the AI-generated sustained-release lornoxicam tablets [43]. Dissolution studies were conducted in 900 mL of phosphate buffer pH 6.8 maintained at 37 ± 0.5 °C with a paddle rotation speed of 50 rpm. This medium was selected to represent intestinal pH conditions relevant to lornoxicam release and to ensure adequate drug solubility. At the nominal dose of 16 mg per tablet dissolved in 900 mL, the maximum theoretical drug concentration at complete release is approximately 17.8 µg/mL, which is well below one-third of the reported aqueous solubility of lornoxicam at pH 6.8 (approximately 150 µg/mL), confirming that sink conditions were maintained throughout the study [8]. Although the AI models were initially prompted to target a 12 h release profile, preliminary dissolution testing of the reference product (LOROX OD 16 mg) revealed continued drug release beyond 12 h; accordingly, the dissolution study was extended to 24 h to fully characterize the release profiles of both the reference and AI-generated formulations. At predetermined time intervals (0.5, 1, 2, 3, 4, 6, 8, 10, 12 and 24 h), 5 mL samples were withdrawn, filtered through 0.45 μm mixed cellulose ester (MCE) membrane filters (13 mm, Merck Millipore), and analysed by UV visible spectrophotometry at 378 nm [45]. An equal volume of fresh medium was replaced after each sampling to maintain constant volume and sink conditions. Six tablets from each formulation were tested (n = 6), and results were expressed as mean cumulative percentage drug released ± standard deviation. Dissolution profiles were constructed by plotting cumulative percent drug released versus time and were subsequently used for similarity analysis and kinetic modelling [46]. Dissolution results were interpreted in conjunction with formulation composition and physical tablet properties.

### 4.6. Similarity and Difference Factor Analysis

Dissolution profile similarity was assessed using difference factor (f_1_) and similarity factor (f_2_) calculated according to regulatory guidelines:

(1) $$f_{1}=\left\{\frac{\sum_{t=1}^{n}|R_{t}-T_{t}|}{\sum_{t=1}^{n}R_{t}}\right\}\times 100$$

(2) $$f_{2}=50\times \log\{{\left[1+\frac{1}{n}\overset{n}{\underset{t=1}{\sum }}{\left(R_{t}-T_{t}\right)}^{2}\right]}^{-0.5}|100\}$$

where n is the number of time points, Rt is the percent dissolved of the reference profile at time t, and Tt is the percent dissolved of the test profile at time t [47].

According to regulatory criteria, f_1_ values below 15 and f_2_ values between 50 and 100 indicate profile similarity [47]. In this study, the innovator product was used as the reference profile for comparison. In accordance with regulatory requirements, only one time point beyond 85% cumulative release was included in the f_1_ and f_2_ calculations for each formulation pair [46,47]. The f_1_ and f_2_ values were applied to rank the AI-generated formulations relative to the reference and were interpreted alongside visual inspection of the dissolution curves to provide integrated assessment of release behaviour [19].

### 4.7. Release Kinetics Modeling

Dissolution data were fitted to commonly used kinetic models to characterize drug release behaviour and explore potential release mechanisms from hydrophilic matrix systems [33,48]. The applied models included zero order, first order, Higuchi, and Korsmeyer–Peppas models.

Zero order kinetics was evaluated according to:

*Qt = Q*_0_*+ K*_0_*t* (3)

where Qt is the cumulative amount of drug released at time t, Q_0_ is the initial amount, and K_0_ is the zero-order release constant [33]. Zero order release describes concentration independent drug release over time.

First order kinetics was evaluated using the logarithm of the remaining drug fraction as a function of time:

*ln(Q*_0_ *− Qt) = ln Q*_0_ *− K*_1_*t* (4)

Q_0_ represents the initial amount of drug in the dosage form, Q_t_ is the cumulative amount of drug released at time t, and K_1_ is the first order release constant, describing concentration dependent release behaviour [49].

The Higuchi model was applied according to:

*Qt = K_h_√t* (5)

where K_h_ is the Higuchi dissolution constant, typically associated with diffusion-controlled drug release from porous matrices [50].

The Korsmeyer–Peppas model was applied to the initial phase of release (up to 60 percent drug release) using:

*Mt/M∞ = Ktⁿ* (6)

where Mt/M∞ is the fraction of drug released at time t, K is the release rate constant, and n is the release exponent [33]. The value of n provides insight into the dominant transport mechanism: n ≈ 0.45 indicates Fickian diffusion; 0.45 < n < 0.89 indicates anomalous transport involving combined diffusion and polymer relaxation; and n ≥ 0.89 suggests Case II or super case II transport dominated by polymer relaxation processes [51]. For comparative purposes, the model exhibiting the highest R^2^ value was considered descriptively representative for each formulation. However, kinetic parameters were interpreted cautiously by considering model assumptions and consistency with observed dissolution profiles, recognizing that hydrophilic matrix tablets exhibit complex release behaviour involving simultaneous diffusion, swelling, erosion, and structural relaxation processes [52].

### 4.8. Statistical Analysis

Statistical analyses were performed using the Python programming language (Python version 3.9.0). Data manipulation and organization were conducted using the Pandas library, while numerical computations and statistical analyses were performed using NumPy and SciPy. Graphical visualizations were generated using Matplotlib version 3.10. Pearson correlation coefficients (r) were calculated to evaluate the association between the dissolution similarity factor (f_2_) and the Korsmeyer–Peppas kinetic parameters, including the release rate constant (k_k_), release exponent (n), and goodness-of-fit (R^2^). Correlation strength was interpreted descriptively due to the limited number of formulations evaluated.

Agreement between AI-predicted and experimentally measured dissolution profiles was assessed for each formulation using several predictive accuracy metrics. The root mean square error (RMSE) and mean absolute percentage error (MAPE) were calculated according to the following equations:

*RMSE* = *√*[(1/*n*) *Σ* (*P_i_* − *E_i_*)^2^] (7)

*MAPE* = (100/*n*) *Σ* |(*P_i_* − *E_i_*)/*E_i_*| (8)

where P_i_ and E_i_ represent the AI-predicted and experimentally measured cumulative percentage drug release at time point i, respectively, and n is the number of sampling time points. Mean prediction bias (Bias) was defined as the arithmetic mean of the differences between predicted and experimental values:

*Bias* = (1/*n*) *Σ* (*P_i_* − *E_i_*) (9)

Bland–Altman analysis was performed to evaluate the agreement between predicted and experimental dissolution data and to identify any systematic bias. The mean difference and the 95% limits of agreement (mean difference ±1.96 standard deviations) were calculated and assessed across all sampling time points.

Hierarchical cluster analysis (HCA) was conducted to explore similarity patterns among formulations based on standardized variables (z-score normalization) of the dissolution similarity factor (f_2_) and the Korsmeyer–Peppas parameters (k_k_ and n). Euclidean distance was used as the similarity metric, and Ward’s linkage method was applied to construct the clustering hierarchy. Given the limited dataset, cluster interpretation was considered exploratory and descriptive.

## 5. Conclusions

AI-generated sustained-release lornoxicam matrix formulations were successfully prepared using direct compression and demonstrated controlled-release behaviour consistent with hydrophilic matrix systems. Dissolution, similarity analysis, and kinetic modelling collectively indicated that drug release mechanisms ranged from anomalous transport, reflecting combined diffusion and polymer relaxation, to super Case II transport dominated by polymer relaxation, consistent with the varied polymer compositions across formulations. While several formulations achieved acceptable physical performance, variability in drug content observed in selected DeepSeek formulations highlights the need for further formulation optimization and process control.

These findings support the use of large language models as tools for early-stage formulation ideation and structured exploration of excipient combinations. However, the quantitative prediction of dissolution profiles was limited, as reflected by relatively high RMSE and MAPE values. Therefore, AI-generated dissolution predictions should be interpreted as qualitative guidance rather than accurate numerical forecasts. The present study was not designed to evaluate full formulation optimization or to compare AI-assisted formulation development with conventional formulation strategies. Thus, expert review of AI-generated compositions, mechanistic interpretation, process optimization, and experimental validation remain essential in pharmaceutical development.

## Figures and Tables

**Table 1:** Physical properties of AI-generated lornoxicam sustained-release tablet formulations and reference product.

Formulation	Weight (mg)	Hardness (N)	Friability (%)	Drug Content (%)
Reference product	243.47	101.1	0.826	—
F1C	442.77	123.4	0.225	95.34
F2C	444.23	102.9	0.000	95.74
F3C	451.90	68.8	1.108	103.00
F4D	402.32	139.5	0.000	97.00
F5D	402.92	132.0	0.247	119.38
F6D	399.96	102.9	0.249	116.56

—: drug content not tested

**Table 2:** Comparison of dissolution profiles of the test formulations with the reference product using difference factor (f_1_) and similarity factor (f_2_).

Compared Formulae	f1 Value	f2 Value
R vs. F1C	47.33	28.61
R vs.F2C	25.30	49.70
R vs.F3C	9.66	71.31
R vs.F4D	27.79	48.06
R vs.F5D	24.36	49.16
R vs.F6D	67.97	31.98

**Table 3:** Correlation coefficients (R^2^) for release data of lornoxicam from different formulations after curve fitting to zero-order, first-order, Higuchi, and Korsmeyer–Peppas (K–P) models. kk = ln(K), where K is the release rate constant; n = release exponent.

	Zero Order Model		First Order Model		Higuchi Model		Korsmeyer Peppas			
Code	K_0_	R^2^	K_1_	R^2^	K_H_	R^2^	k_k_	R^2^	N	Drug Transport Mechanism
R	0.0700	0.98	−0.0040	0.75	2.5188	0.80	−1.8217	0.99	1.17	Super Case II transport
F1C	0.0333	0.99	−0.0002	0.99	1.2610	0.89	−1.8429	0.98	1.13	Super Case II transport
F2C	0.0719	0.99	−0.0040	0.77	2.7079	0.88	−1.7068	0.99	1.20	Super Case II transport
F3C	0.0719	0.98	−0.0040	0.75	2.5628	0.78	−2.7756	0.99	1.53	Super Case II transport
F4D	0.0637	0.99	−0.0009	0.88	2.4047	0.89	−0.6375	0.99	0.81	Anomalous (non-Fickian) diffusion
F5D	0.0731	0.98	−0.0040	0.77	2.7868	0.89	−2.1198	0.96	1.35	Super Case II transport
F6D	0.0620	0.92	−0.0012	0.91	2.5666	0.99	0.2774	0.99	0.54	Anomalous (non-Fickian) diffusion

**Table 4:** Similarity factors (f_2_) versus the reference product and Korsmeyer–Peppas (K–P) kinetic parameters: release rate constant (k_k_) and release exponent (n).

Formulation	f_2_	k_k_	n	R^2^_kp_
F1C	28.61	−1.8429	1.13	0.98
F2C	49.70	−1.7068	1.20	0.99
F3C	71.31	−2.7756	1.53	0.99
F4D	48.06	−0.6375	0.81	0.99
F5D	49.16	−2.1198	1.35	0.96
F6D	31.98	0.2774	0.54	0.99

Kinetic parameters were derived from regression analysis performed on mean dissolution profiles (n = 6 vessels per formulation).

**Table 5:** Formulation-level and mean prediction accuracy metrics comparing AI-predicted and experimentally measured dissolution profiles of lornoxicam sustained-release formulations.

AI-Model	Formulation	RMSE (%)	MAPE (%)	Bias	Pearson r
ChatGPT	F1C	17.9	45	Negative	0.97
ChatGPT	F2C	22.6	38	Positive	0.99
ChatGPT	F3C	24.3	46	Positive	0.99
ChatGPT (Mean)		≈21.6	≈43		≈0.98
DeepSeek	F4D	21.3	52	Positive	0.96
DeepSeek	F5D	25.7	60	Positive	0.95
DeepSeek	F6D	19.8	48	Positive	0.97
DeepSeek (Mean)		≈22.3	≈53		≈0.96

RMSE: Root mean square error; MAPE: Mean absolute percentage error; Bias: Direction of systematic prediction error; r: Pearson correlation coefficient. Mean values represent the arithmetic average of the corresponding formulation-level metrics.

**Table 6:** Cluster membership of AI-generated formulations based on hierarchical cluster analysis.

Cluster	Formulations	General Characteristics
1	F3C, F5D	Higher n; divergent f_2_
2	F2C, F4D	Moderate f_2_
3	F1C, F6D	Lower f_2_, lower n

**Table 7:** List of suggested excipients and their roles for AI models to choose from.

Materials	Role
Lornoxicam	Active ingredient
HPMC K4M DC	These polymers matrix are the family of hydroxypropyl methyl cellulose with different molecular weight and different viscosities. The high molecular weight means high viscosity and longer releases of the drug.
HPMC K100M DC	
HPMC F50	
HPMC E4M	
HPMC A15LV	
HPMC E4M CR	
Carbopol 71G	Polyacrylic acid polymer matrix
Croscarmellose Sodium	Disintegrant to facilitate drug release
Crosspovidone XL-10	
Microcrystalline cellulose	Binders to hold the tablet together
Plasdone K-29/32	
Dicalcium phosphate	
Starch	
Mannitol DC	
Magnesium Stearate	Glidant and lubricant
Talc	
Colloidal Silicon Dioxide	

**Table 8:** Composition of AI-generated lornoxicam extended-release matrix tablet formulations (tablet weight indicated in column headers).

Component	F1C (430 mg)	F2C (430 mg)	F3C (430 mg)	F4D (400 mg)	F5D (400 mg)	F6D (400 mg)
Lornoxicam	16 mg (3.7%)	16 mg (3.7%)	16 mg (3.7%)	16 mg (4.0%)	16 mg (4.0%)	16 mg (4.0%)
HPMC K100M DC	160 mg (37.2%)	–	–	50 mg (12.5%)	–	70 mg (17.5%)
HPMC K4M DC	–	100 mg (23.3%)	–	20 mg (5.0%)	–	–
HPMC E4M	–	–	100 mg (23.3%)	–	–	–
HPMC E4M CR	–	60 mg (14.0%)	–	–	40 mg (10.0%)	–
Carbopol 71G	–	–	40 mg (9.3%)	–	30 mg (7.5%)	–
Microcrystalline Cellulose	130 mg (30.2%)	–	–	250 mg (62.5%)	240 mg (60.0%)	230 mg (57.5%)
Plasdone K-29/32	–	120 mg (27.9%)	–	–	–	–
Dicalcium Phosphate	–	–	140 mg (32.6%)	–	–	–
Mannitol DC	100 mg (23.3%)	110 mg (25.6%)	110 mg (25.6%)	60 mg (15.0%)	70 mg (17.5%)	76 mg (19.0%)
Crosspovidone XL-10	20 mg (4.7%)	–	20 mg (4.7%)	–	–	–
Croscarmellose Sodium	–	20 mg (4.7%)	–	–	–	4 mg (1.0%)
Colloidal Silicon Dioxide	–	–	–	1 mg (0.25%)	1 mg (0.25%)	1 mg (0.25%)
Magnesium Stearate	4 mg (0.9%)	4 mg (0.9%)	4 mg (0.9%)	3 mg (0.75%)	3 mg (0.75%)	3 mg (0.75%)

–: not included in the formulation.

## Data Availability

The data presented in this study are available upon reasonable request from the corresponding author.

## Abbreviations

The following abbreviations are used in this manuscript:

AI	Artificial intelligence
BCS	Biopharmaceutics Classification System
DCP	Dicalcium phosphate
HCA	Hierarchical cluster analysis
HPMC	Hydroxypropyl methylcellulose
LLM	Large language model
MAPE	Mean absolute percentage error
MCE	Mixed cellulose ester
RMSE	Root mean square error
USP	United States Pharmacopeia
